# Bis[μ-bis­(2,6-diiso­propyl­phen­yl) phosphato-κ^2^ *O*:*O*′]bis­[(2,2′-bi­pyridine-κ^2^ *N*,*N*′)lithium] toluene disolvate and its catalytic activity in ring-opening polymerization of ∊-caprolactone and l-dilactide

**DOI:** 10.1107/S2056989019006960

**Published:** 2019-05-21

**Authors:** Alexey E. Kalugin, Pavel D. Komarov, Mikhail E. Minyaev, Konstantin A. Lyssenko, Dmitrii M. Roitershtein, Ilya E. Nifant’ev

**Affiliations:** a A.V. Topchiev Institute of Petrochemical Synthesis, Russian Academy of Sciences, 29 Leninsky Prospect, 119991, Moscow, Russian Federation; b Moscow Institute of Physics and Technology, Department of Biological and Medical Physics, 9 Institutskiy Per., Dolgoprudny, Moscow Region, 141701, Russian Federation; c G.V. Plekhanov Russian University of Economics, 36 Stremyanny Per., Moscow, 117997, Russian Federation; d Chemistry Department, M.V. Lomonosov Moscow State University, 1 Leninskie Gory Str., Building 3, Moscow 119991, Russian Federation; e N.D. Zelinsky Institute of Organic Chemistry, Russian Academy of Sciences, 47 Leninsky Prospect, Moscow 119991, Russian Federation

**Keywords:** lithium, diaryl phosphate, bi­pyridine, coordination compound, crystal structure, ring-opening polymerization, cyclic esters

## Abstract

The solvated centrosymmmtric title compound, [Li_2_(C_24_H_34_O_4_P)_2_(C_10_H_8_N_2_)_2_]·2C_7_H_8_, was formed in the reaction between {Li[(2,6-^i^Pr_2_C_6_H_3_-O)_2_POO](MeOH)_3_}(MeOH) and 2,2′-bi­pyridine (bipy) in toluene. The structure has monoclinic (*P*2_1_/*n*) symmetry at 120 K and the asymmetric unit consists of half a complex mol­ecule and one mol­ecule of toluene solvent. The diaryl phosphate ligand demonstrates a μ-κ*O*:κ*O*′-bridging coordination mode and the 2,2′-bi­pyridine ligand is chelating to the Li^+^ cation, generating a distorted tetra­hedral LiN_2_O_2_ coordination polyhedron. The complex exhibits a unique dimeric Li_2_O_4_P_2_ core. One isopropyl group is disordered over two orientations in a 0.621 (4):0.379 (4) ratio. In the crystal, weak C—H⋯O and C—H⋯π inter­actions help to consolidate the packing. Catalytic systems based on the title complex and on the closely related complex {Li[(2,6-^i^Pr_2_C_6_H_3_-O)_2_POO](MeOH)_3_}(MeOH) display activity in the ring-opening polymerization of ∊-caprolactone and l-dilactide.

## Abstract 2

The solvated centrosymmmtric title compound, [Li_2_(C_24_H_34_O_4_P)_2_(C_10_H_8_N_2_)_2_]·2C_7_H_8_, was formed in the reaction between {Li[(2,6-^i^Pr_2_C_6_H_3_-O)_2_POO](MeOH)_3_}(MeOH) and 2,2′-bi­pyridine (bipy) in toluene. The diaryl phosphate ligand demonstrates a μ-κ*O*:κ*O*′-bridging coordination mode and the 2,2′-bi­pyridine ligand is chelating to the Li^+^ cation generating a distorted tetra­hedral LiN_2_O_2_ coordination polyhedron. The complex exhibits a unique dimeric Li_2_O_4_P_2_ core. Catalytic systems based on the title complex and on the closely related complex {Li[(2,6-^i^Pr_2_C_6_H_3_-O)_2_POO](MeOH)_3_}(MeOH) display activity in the ring-opening polymerization of ∊-caprolactone and l-dilactide.

## Chemical context   

Various *d-* and *f-*metal complexes with disubstituted organophosphate ligands are currently being studied, for example, as model compounds to explore the role of biometals in biological systems, including complexes that mimic the functions, structure and reactivity of active centers of enzymes in order to create models of biologically active metal centers (Kövári & Krämer, 1996 ▸; Lipscomb & Sträter, 1996 ▸; Hegg & Burstyn, 1998 ▸; Hegg *et al.*, 1999 ▸; Atkinson & Lindoy, 2000 ▸; Deck *et al.*, 2002 ▸; Reichenbach-Klinke & König, 2002 ▸; Fry *et al.*, 2005 ▸; Dey *et al.*, 2012 ▸; Sato *et al.*, 2012 ▸), as catalysts for various catalytic processes, *e.g*. alkene cyclo­propanation and carbene insertions (Lacasse *et al.*, 2005 ▸; Hrdina *et al.*, 2013 ▸), polymerization of conjugated dienes (Anwander, 2002 ▸; Friebe *et al.*, 2006 ▸; Kobayashi & Anwander, 2001 ▸; Minyaev *et al.*, 2018*a* ▸,*b* ▸,*c* ▸; Nifant’ev *et al.*, 2013 ▸, 2014 ▸; Zhang *et al.*, 2010 ▸) acrylo­nitrile (Minyaev *et al.*, 2018*d* ▸) and dilactide (Nifant’ev *et al.*, 2013 ▸) and inhibition of thermal decomposition of polydi­methyl­siloxanes (Minyaev *et al.*, 2018*a* ▸,*e* ▸). Synthetic precursors for these com­plexes are the corresponding alkali metal organophos­phate derivatives, whose structures are still poorly explored.
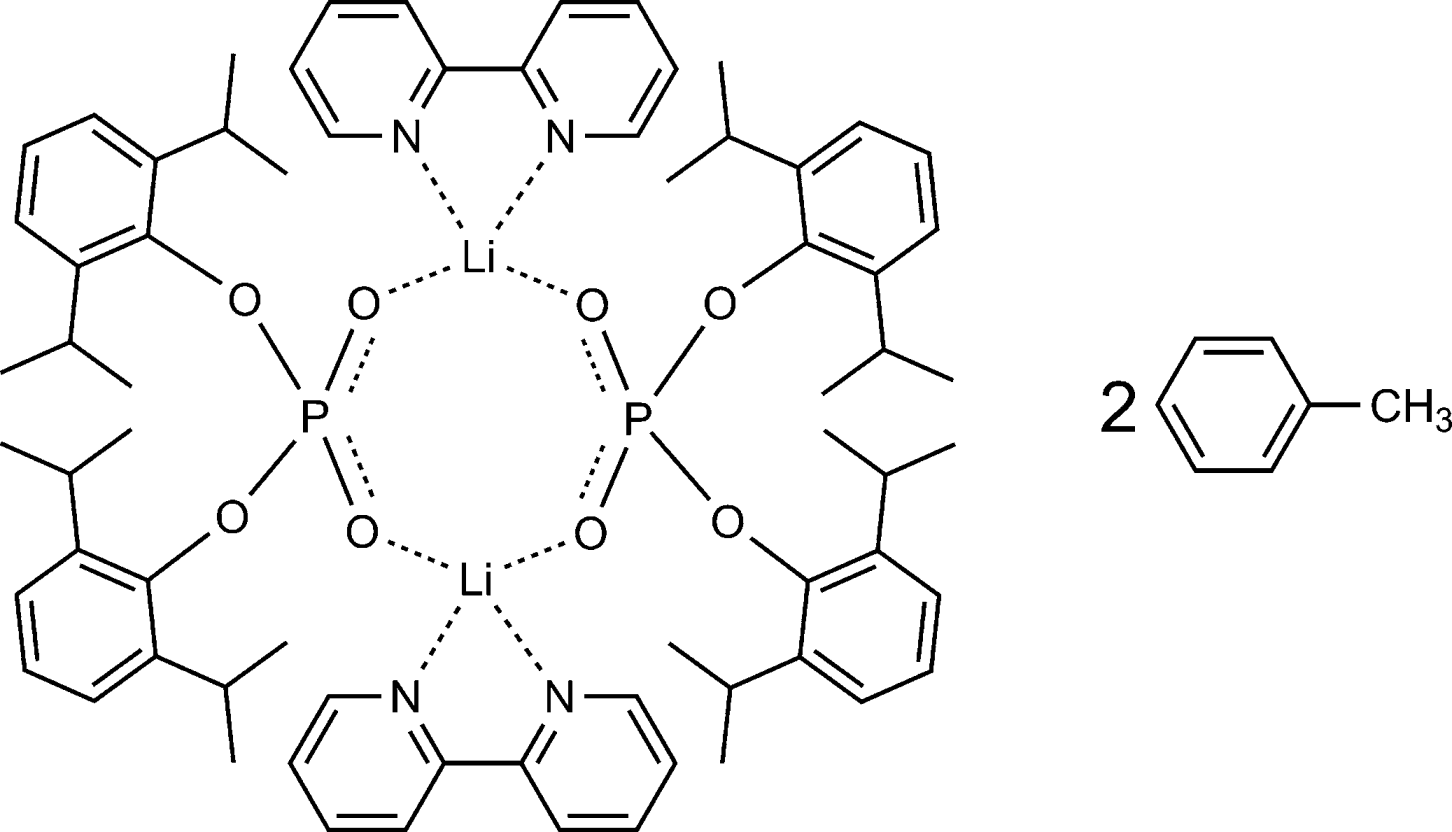


**Figure 1:**
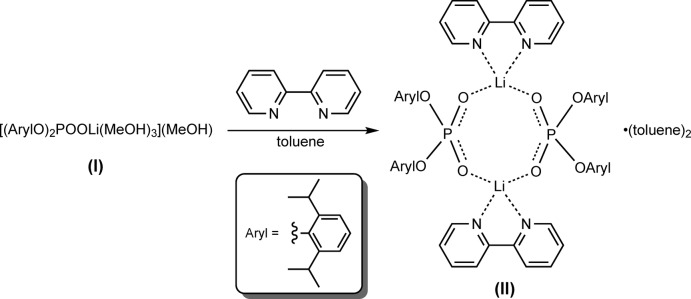
Synthesis of {[(2,6-^*i*^Pr_2_C_6_H_3_O)_2_PO_2_]Li(bipy)}_2_(C_7_H_8_)_2_, (II).

Recently we have reported on the structure of the lithium salt {Li[(2,6-^i^Pr_2_C_6_H_3_-O)_2_POO](MeOH)_3_}(MeOH), (I), having the same ligand (Minyaev *et al.*, 2015 ▸) as in the title compound. Attempts to use this salt to produce Tb and Eu phosphate complexes with luminescent properties have led to complexes having coordinated methanol mol­ecules and possessing very low quantum yields (unpublished results). The presence of the MeOH mol­ecules and therefore undesirable *Ln*—O—H bonds usually noticeably decreases the quantum yield because of quenching luminescence (Bünzli & Piguet, 2005 ▸; Bünzli, 2017 ▸; Yan *et al.*, 1995 ▸; Sy *et al.*, 2016 ▸). At the same time, the phosphate ligand in the complexes has not displayed properties of an ‘antenna’ ligand for luminescence sensitization (Bünzli & Piguet, 2005 ▸; Bünzli *et al.*, 2007 ▸; Sy *et al.*, 2016 ▸; Guillou *et al.*, 2016 ▸; Hewitt & Butler, 2018 ▸; Roitershtein *et al.*, 2018 ▸). A 2,2′-bi­pyridine (bipy) mol­ecule can serve as such an ‘antenna’, which usually increases the quantum yield dramatically. We have found that salt (I) can be easily converted into the complex {Li_2_(bipy-κ^2^ *N*,*N*′)_2_[(2,6-^*i*^Pr_2_C_6_H_3_-O)_2_POO-μ-κ*O*:κ*O*′]_2_}(C_7_H_8_)_2_ (II) (Fig. 1 ▸), the crystal structure of which is reported herein. It has no coordinated MeOH mol­ecule, but has the ‘built-in’ bipy ligand. Therefore, complex (II) might be successfully utilized in the synthesis of luminescent rare-earth organophosphate complexes.

On the other hand, it is known that diaryl-substituted phospho­ric acids in the presence of 3-phenyl­propan-1-ol as an initiator are capable of catalysing ring-opening polymerization (ROP) of ∊-caprolactone (∊-CL) and l-lactide (LLA) into poly(∊-caprolactone) (PCL) and poly(l-lactide) (PLLA) at high temperatures (453 K, bulk sample; Liu *et al.*, 2019 ▸). It might be noted that ROP of ∊-CL and LLA can also be carried out at lower temperatures: 353 K for ∊-CL (bulk sample, the same initiator and catalysts; Saito *et al.*, 2015 ▸) and 383 K for dl-lactide [30% of toluene by volume, glycolic acid derivatives of bio-metals (Mg, Zn, Al) were used as catalysts; Nifant’ev *et al.*, 2018 ▸].

**Figure 2:**
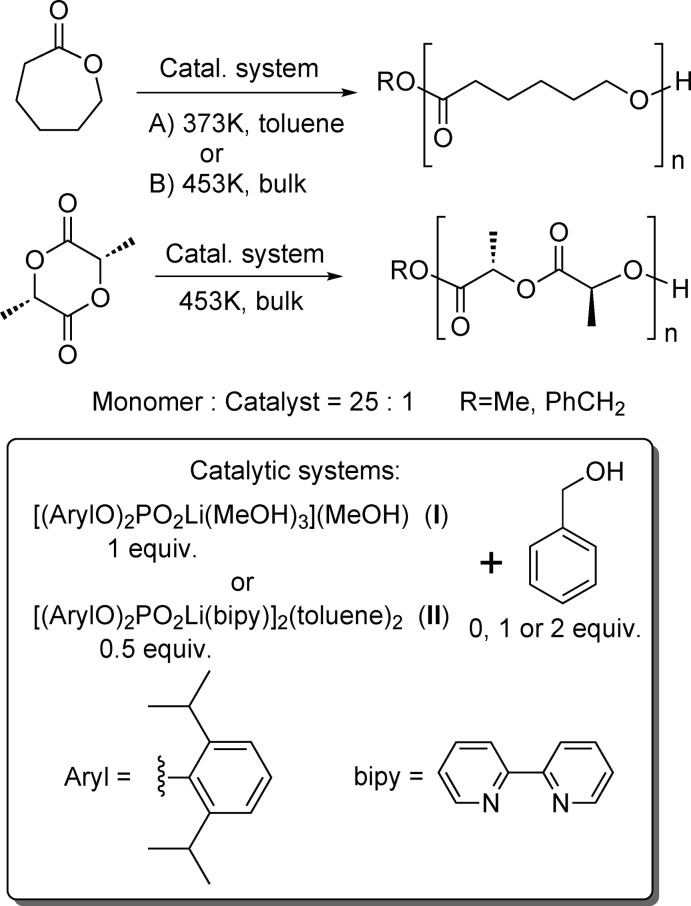
Ring-opening polymerization of ∊-caprolactone and l-dilactide using complexes {[(2,6-^*i*^Pr_2_C_6_H_3_O)_2_PO_2_]Li(MeOH)_3_}(MeOH), (I), and {[(2,6-^i^Pr_2_C_6_H_3_O)_2_PO_2_]Li(bipy)}_2_(C_7_H_8_)_2_, (II).

We have tested salts (I) and (II) as precatalysts for ∊-CL and LLA polymerization under two different condition sets: (1) 373 K, ∼30% of toluene by volume and (2) 453 K, bulk sample (Fig. 2 ▸), using benzyl alcohol as an initiator. The monomer/precatalyst molar ratio is taken as 25:1 (with respect to one lithium phosphate unit) in order to monitor the reaction mixtures and to study the resulting short oligomers by ^1^H NMR spectroscopy.

The ROP of ∊-CL catalysed by (I) proceeds at different equivalents of an activator under mild conditions, providing PCL with a high conversion of ∊-CL (Table 1 ▸, entries 1–3). The 100% conversion and a higher polymerization degree (*P_n_*), which is a number of oligomerized monomer units, has been observed in the presence of two equivalents of PhCH_2_OH activator (entry 3). However, even in the absence of PhCH_2_OH (entry 1), the MeOH mol­ecules in complex (I) act as an activator as well. According to the ^1^H NMR data for PCL obtained, there are two types of the RO terminal group, namely, MeO and PhCH_2_O. Based on NMR integral intensities, their sum corresponds to the amount of the –CH_2_—OH terminal group. The MeO/PhCH_2_O ratio decreases upon increasing the taken amount of PhCH_2_OH, and the corres­ponding ratio is 1.00/0.00 for entry 1, 0.73/0.27 for entry 2 and 0.58/0.42 for entry 3. Thus, compound (I) does not require an additional activator because of the presence of the inter­nal one, namely, MeOH mol­ecules. Unlike for (I), polymerization of ∊-CL by (II) without an activator does not occur (entry 4). Activation by benzyl alcohol does not lead to a noticeable yield of PCL having only the PhCH_2_O– and –CH_2_—OH terminal groups (entries 5 and 6). The addition of two equivalents of the PhCH_2_OH activator provides higher conversions in the cases of both complexes (I) and (II) (entries 3 and 6).

Unlike ∊-CL oligomerization, the ROP of LLA has failed under the same conditions. For example, conversion of LLA to PLLA at the [LLA]/[(II)]/[PhCH_2_OH] ratio of 25:0.5:2 is only 6%. Therefore, oligomerization of LLA and ∊-CL has been studied further at a higher temperature (Table 2 ▸). Conversions of ∊-CL in the case of complex (II) (entry 2) is even higher than that for (I), but the polymerization degree is higher than expected *P_n_* = 25, which may be explained by the faster reaction rate of the catalyst with the monomer, compared to the activation rate. The ROP of LLA proceeds under these conditions, but providing a rather low conversion to PLLA and the formation of shorter oligomers than expected (entries 3 and 4).

In summary, catalytic tests have displayed that systems based on complexes (I) and (II) are capable of catalysing ROP of cyclic esters, using ∊-caprolactone and l-dilactide as model substrates, but the catalytic activity of the systems is rather poor. Complex (I) does not require an initiator for polymerization of ∊-CL.

## Structural commentary   

**Figure 3:**
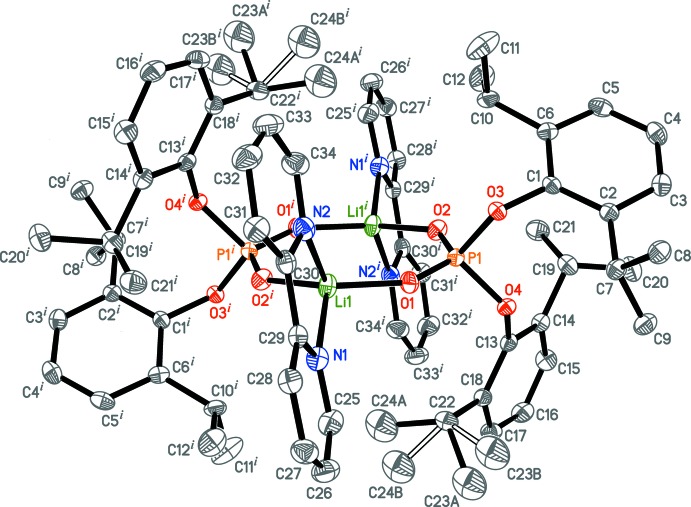
The mol­ecular structure of {Li_2_(bipy)_2_[(2,6-^*i*^Pr_2_C_6_H_3_-O)_2_PO_2_]_2_}. Displacement ellipsoids are drawn at the 50% probability level. Hydrogen atoms are omitted for clarity. The minor disorder component of one of the isopropyl groups is shown with open lines. Symmetry code: (i) −*x* + 1, −*y* + 1, −*z* + 2.

The title compound (II) crystallizes in the monoclinic space group (*P*2_1_/*n*). Its asymmetric unit (see Fig. S1 in the supporting information) contains one non-coordinating toluene mol­ecule and half the complex {Li_2_(bipy)_2_[(2,6-^*i*^Pr_2_C_6_H_3_-O)_2_PO_2_]_2_}, which is located on an inversion centre (Fig. 3 ▸) [symmetry code: (i) −*x* + 1, −*y* + 1, −*z* + 2]. The complex has an unusual Li_2_P_2_O_4_ core (Fig. S2 in the supporting information), in which all the O and P atoms lie in the same plane, but the Li atoms deviate from the plane by 0.338 (4) Å. According to the Cambridge Structural Database (CSD version 5.40 with updates, Groom *et al.*, 2016 ▸), there are 27 crystal structures of alkali metal derivatives with (*R* ^1^O)(*R* ^2^O)PO_2_^−^ anions (*R* ^1^, *R* ^2^ = alkyl, ar­yl). Only the two-dimensional coordination polymer structure of Na[O_2_P(O-C_6_H_4_-4-NO_2_)_2_] (CSD refcodes AGACIW/AGACIW01; Gerus & Lis, 2013 ▸; Starynowicz & Lis, 2014 ▸) displays an Na_2_P_2_O_4_ structural motif similar to the Li_2_P_2_O_4_ core found in (II). On the other hand, there are a number of lithium carboxyl­ates demonstrating a very similar Li_2_C_2_O_4_ core with the *R*CO_2_^−^ ligand in the same μ_2_-κ*O*:κ*O*′-bridging mode (see the CSD).

The [Li(bipy)]^+^ cation in (II) is nearly flat with the highest deviations from the plane being 0.102 (2) Å for N2, 0.115 (2) Å for C31 and 0.133 (2) Å for C34. To be more precise, the coordinated bipy ligand is slightly twisted about the C29—C30 bond; the dihedral angle between two planes formed by the N1/C25–C29 and N2/C30–C34 atoms is 8.41 (12)°. Selected bond distances are given in Table 3 ▸. The P—O_Ar_ bond distances are longer by 0.13–0.14 Å than the other two P—O distances. The P and Li atoms adopt distorted tetra­hedral environments with the bond angles ranging from 77.49 (14)° for N1—Li1—N2 to 120.5 (2)° for O1—Li1—O2^i^ and from 98.16 (8)° for O3—P1—O4 to 120.32 (9)° for O1—P1—O2. The smallest O—P—O angle corresponds to the O_Ar_—P—O_Ar_ angle between the two bulky aryl ligands. These observations for the P—O distances and O—P—O bond angles are also seen for the closely related salt (I) (Minyaev *et al.*, 2015 ▸), for rare-earth complexes bearing the same phosphate ligand (Minyaev *et al.*, 2017 ▸, 2018*a* ▸,*b* ▸,*c* ▸) and for bis­(2,6-diiso­propyl­phen­yl)phospho­ric acid (with the exception of the P—OH bond-distance value; Gupta *et al.*, 2018 ▸). An explan­ation for this has been given earlier (Minyaev *et al.*, 2017 ▸).

## Supra­molecular features   

The extended structure of (II), for which packing plots are shown in Figs. S3–S5 of the supporting information, features weak C—H⋯O and C—H⋯π inter­actions (Table 4 ▸). Any aromatic π–π stacking must be extremely weak, as the shortest centroid–centroid separation of aromatic rings is 4.1743 (13) Å.

## Synthesis and crystallization   

### General remarks   

All synthetic manipulations were performed under a purified argon atmosphere, using Schlenk glassware, dry box techniques and absolute solvents. Hexane was distilled from Na/K alloy, toluene was distilled from sodium/benzo­phenone ketyl, 2,2′-bi­pyridine was recrystallized from absolute toluene prior to use. The salt [(2,6-^*i*^Pr_2_C_6_H_3_O)_2_PO_2_Li(MeOH)_3_](MeOH) was synthesized according to the literature procedure (Minyaev *et al.*, 2015 ▸). (3*S*,6*S*)-3,6-Dimethyl-1,4-dioxane-2,5-dione (l-lactide, Sigma–Aldrich, 99%) was purified by double sublimation under dynamic vacuum. ∊-Caprolactone (∊-CL) was distilled from CaH_2_ under vacuum. CDCl_3_ (Cambridge Isotope Laboratories, Inc., D 99.8%) was used as purchased for registering the NMR spectra of polymer samples, and was distilled from CaH_2_ under argon prior to recording the NMR spectra of (II). The ^1^H NMR spectra of polymers were recorded on a Bruker AVANCE 400 spectrometer at 297K, the ^1^H and ^31^P{^1^H} NMR spectra of (II) were registered on a Bruker AV-600 instrument; chemical shifts are reported in ppm relative to the solvent residual peak. Size-exclusion chromatography (SEC) analysis of polymer samples was performed at 323 K using an Agilent PL-GPC 220 gel permeation chromatograph equipped with a PLgel column, with DMF as eluents (1 ml min^−1^) and poly(ethyl­ene oxide) standards.

### Synthesis and crystallization of (II)   

A solution of 2,2′-bi­pyridine (187 mg, 1.2 mmol) in toluene (5 ml) was added dropwise to a stirred solution of (2,6-^*i*^Pr_2_C _6_H_3_O)_2_PO_2_Li(MeOH)_4_ (553 mg, 1 mmol) in toluene (20 ml). During the addition of bi­pyridine, the reaction mixture became opaque as a result of the precipitation of microcrystalline {Li_2_(bipy)_2_[(2,6-^*i*^Pr_2_C_6_H_3_O)_2_PO_2_]_2_}(C_7_H_8_)_2_. After the addition was complete, the mixture was stirred for 1 h, and allowed to settle. The resulting solution was deca­nted. The white crystalline solid was washed with hexane (2 × 3 ml) and dried under dynamic vacuum. The yield was 84% (565 mg, 0.42 mmol) of white solid. ^1^H NMR (600 MHz, CDCl_3_): δ = 0.95 [48H, *d*, ^3^ *J*_HH_ =6.84Hz, –CH(C***H_3_***)_2_], 2.37 (6H, *s*, C_6_H_5_—C***H_3_***), 3.59 [8H, *septet*, *-*-C***H***(CH_3_)_2_], 6.93–6.97 (12H, *m*, O^*i*^Pr_2_C_6_ ***H_3_***), 7.17 (2H, *t*, ***H_para_*** in C_6_H_5_—CH_3_), 7.19 (4H, *d*, ***H_ortho_*** in C_6_H_5_—CH_3_), 7.20 (4H, *dd*, ^3^ *J*_HH_ = 5.25Hz and 7.27Hz, ***H5***-bipy), 7.26 (4H, *t*, ***H_meta_*** in C_6_H_5_—CH_3_), 7.77 (4H, *t*, ***H4***-bipy), 8.09 (4H, *d*, ^3^ *J*_HH_ = 8.00Hz, ***H3***-bipy), 8.34 (4H, *d*, ^3^ *J*_HH_ = 3.76Hz, ***H6***-bipy). ^31^P{^1^H} NMR (242.9 MHz, CDCl_3_): δ = 10.82. According to ^1^H NMR data, prolonged vacuum drying of (II) may lead to a nearly complete loss of the non-coord­inating toluene mol­ecules.

Colourless prisms of (II) were formed upon recrystallization of the obtained microcrystalline solid from a warm (∼333 K) nearly saturated solution in toluene by slow cooling to ∼268 K.

### Polymerization procedures   

**Method 1.** In a dry box, complex (I) (0.1 mmol, 55 mg) or complex (II) (0.05 mmol, 67 mg), a monomer (2.5 mmol, 285 mg for ∊-CL or 360 mg for LLA), and toluene (0.15 ml) or a solution of PhCH_2_OH (11 or 22 mg) in toluene (0.15 ml) were placed at room temperature (∼298 K) in a vial, which was then sealed and taken out of the box. The mixture was stirred for 3 h at 373 K. After that, a sample of the mixture was taken to register a ^1^H NMR spectrum to determine the monomer conversion. The remaining mixture was quenched with methanol (tenfold volume) containing 5 equiv. of acetic acid (with respect to Li phosphate), washed with methanol, dried under vacuum, taken for SEC and ^1^H NMR studies.

**Method 2** was performed as **Method 1** with the following exceptions: (1) toluene was not added, (2) the mixture was stirred for 1 h at 453 K.

The monomer conversion was determined by ^1^H NMR (in CDCl_3_) of reaction mixtures, basing on integration of the following resonance signals: C***H_2_***OC=O at 4.22 ppm for ∊-CL and at 4.05 ppm for PCL, C***H***(CH_3_)OC=O at 5.04 ppm for LLA and at 5.15 ppm for PLLA. The end-group analysis was based on the following resonance signals of terminal-groups: 3.67 ppm for C***H_3_***—O—CO–, 5.11 ppm for Ph—C***H_2_***—O—CO–, 3.63 ppm for –CH_2_–C***H_2_***—OH in PCL and 4.37 ppm for –CO—C***H***CH_3_—OH in PLLA.

## Refinement   

Crystal data, data collection and structure refinement details are summarized in Table 5 ▸. The positions of hydrogen atoms (with the exception of the disordered fragment) were found from a difference-Fourier map but positioned geometrically (C—H distance = 0.95 Å for aromatic, 0.98 Å for methyl and 1.00 Å for methine H atoms) and refined as riding atoms with relative isotropic displacement parameters *U*_iso_(H) = 1.5*U*_eq_(C) for methyl H atoms and 1.2*U*_eq_(C) otherwise. A rotating group model was applied for methyl groups. Reflection 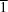
10 was affected by the beam stop and was therefore omitted from the refinement.

One isopropyl group is disordered over two orientations (atoms C23*A*, C24*A* and C23*B*, C24*B*) with a corresponding disorder ratio of 0.621 (4):0.379 (4). Similarity displacement ellipsoid constraints were applied for these atoms. The C—C bond distances in the disordered isopropyl fragment were restrained to be equal within 0.002 Å.

## Supplementary Material

Crystal structure: contains datablock(s) I. DOI: 10.1107/S2056989019006960/hb7820sup1.cif

Structure factors: contains datablock(s) I. DOI: 10.1107/S2056989019006960/hb7820Isup2.hkl

Supporting information file. DOI: 10.1107/S2056989019006960/hb7820Isup3.cdx

supporting information. DOI: 10.1107/S2056989019006960/hb7820sup4.doc

CCDC reference: 1915965

Additional supporting information:  crystallographic information; 3D view; checkCIF report

## Figures and Tables

**Table 1:** Polymerization of ∊-CL under mild conditions *M*_n_ is the number average molar mass; *Đ* is the polydispersity index; *P_n_* is the polymerization degree; *Conv.* is conversion of ∊-CL into PCL and defined as [PCL]/([∊-CL]+[PCL]). Conditions: [∊-CL]/[complex]/[PhCH_2_OH] = 25:1 for (I) and 0.5 for (II): 0–2; toluene volume is 30%; *T* = 373 K, time = 3 h.

Entry	Complex	Equiv. of PhCH_2_OH	*M_n_*, ·10^3^ *^*a*^*	*Ð^*a*^*	*P_n_^*a*^*	*Conv.^*b*^* (%)	*M_n_*, ·10^3^ *^*b*^*	*P_n_^*b*^*
1	(I)	0	2.35	1.09	20	72	2.20	19
2	(I)	1	1.54	1.15	13	72	2.09	18
3	(I)	2	2.10	1.17	18	100	2.57	22
4	(II)	0	–	–	–	0	–	–
5	(II)	1	1.84	1.02	15	25	1.71	14
6	(II)	2	1.36	1.12	11	41	1.82	15

Notes: (*a*) found by size-exclusion chromatography (SEC) measurements; (*b*) determined by ^1^H NMR studies. *M_n_* and *P_n_* were calculated based on the end-group analysis.

**Table 2:** Bulk polymerization of ∊-CL and LLA Conditions: [monomer]/[complex]/[PhCH_2_OH] = 25:1:2 for (I) and 25:0.5:2 for (II); no solvent; *T* = 453 K, time = 1 h.

Entry	Complex	Monomer	*M_n_*, ·10^3^ *^*a*^*	*Ð^*a*^*	*P_n_^*a*^*	*Conv.^*b*^* (%)	*M_n_*, ·10^3^ *^*b*^*	*P_n_^*b*^*
1	(I)	∊-CL	0.85	1.05	7	26	0.69	6
2	(II)	∊-CL	3.79	1.27	32	73	4.21	36
3	(I)	LLA	1.79	1.12	12	62	1.55	10
4	(II)	LLA	2.03	1.18	13	45	2.27	15

Notes: (*a*) found by SEC measurements; (*b*) determined by ^1^H NMR studies.

**Table 3:** Selected bond lengths (Å)

Li1—O1	1.873 (4)	P1—O1	1.4795 (15)
Li1—O2^i^	1.911 (4)	P1—O2	1.4846 (15)
Li1—N1	2.147 (4)	P1—O3	1.6198 (15)
Li1—N2	2.119 (4)	P1—O4	1.6132 (14)

Symmetry code: (i) 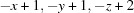.

**Table 4:** Hydrogen-bond geometry (Å, °) *Cg*1 is the centroid of N1/C25–C29, *Cg*2 is the centroid of N2/C30–C34, *Cg*3 is the centroid of C1–C6 and *Cg*5 is the centroid of C35–C40.

*D*—H⋯*A*	*D*—H	H⋯*A*	*D*⋯*A*	*D*—H⋯*A*
C28—H28⋯O3^ii^	0.95	2.60	3.295 (3)	130
C8—H8*C*⋯*Cg*5	0.98	2.60	3.502 (3)	152
C19—H19⋯*Cg*3	1.00	2.73	3.630 (2)	150
C41—H41*A*⋯*Cg*1	0.98	2.83	3.450 (4)	122
C26—H26⋯*Cg*2^ii^	0.95	2.94	3.605 (3)	128
C31—H31⋯*Cg*3^ii^	0.95	2.69	3.591 (3)	159

Symmetry code: (ii) 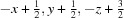.

**Table 5:** Experimental details

Crystal data	
Chemical formula	[Li_2_(C_24_H_34_O_4_P)_2_(C_10_H_8_N_2_)_2_]·2C_7_H_8_
*M*_r_	1345.47
Crystal system, space group	Monoclinic, *P*2_1_/*n*
Temperature (K)	120
*a*, *b*, *c* (Å)	15.2151 (9), 12.9374 (9), 19.5918 (13)
β (°)	106.935 (2)
*V* (Å^3^)	3689.3 (4)
*Z*	2
Radiation type	Mo *K*α
μ (mm^−1^)	0.12
Crystal size (mm)	0.32 × 0.28 × 0.19

Data collection	
Diffractometer	Bruker APEXII CCD
Absorption correction	Multi-scan (*SADABS*; Bruker, 2008 ▸)
*T*_min_, *T*_max_	0.953, 0.990
No. of measured, independent and observed [*I* > 2σ(*I*)] reflections	24668, 11176, 6434
*R*_int_	0.068
(sin θ/λ)_max_ (Å^−1^)	0.714

Refinement	
*R*[*F* ^2^ > 2σ(*F* ^2^)], *wR*(*F* ^2^), *S*	0.065, 0.167, 1.05
No. of reflections	11176
No. of parameters	455
No. of restraints	4
H-atom treatment	H-atom parameters constrained
Δρ_max_, Δρ_min_ (e Å^−3^)	0.73, −0.73

Computer programs: *APEX2* (Bruker, 2008 ▸), *SAINT* (Bruker, 2008 ▸), *SHELXS* (Sheldrick, 2008 ▸), *SHELXL2017* (Sheldrick, 2015*a* ▸), *SHELXTL* (Sheldrick, 2015*b* ▸) and *publCIF* (Westrip, 2010 ▸).

## supplementary crystallographic information

### Crystal data

**Table:**

[Li_2_(C_24_H_34_O_4_P)_2_(C_10_H_8_N_2_)_2_]·2C_7_H_8_	*F*(000) = 1440
*M**_r_* = 1345.47	*D*_x_ = 1.211 Mg m^−^^3^
Monoclinic, *P*2_1_/*n*	Mo *K*α radiation, λ = 0.71073 Å
*a* = 15.2151 (9) Å	Cell parameters from 1860 reflections
*b* = 12.9374 (9) Å	θ = 2.2–22.3°
*c* = 19.5918 (13) Å	µ = 0.12 mm^−^^1^
β = 106.935 (2)°	*T* = 120 K
*V* = 3689.3 (4) Å^3^	Prism, colourless
*Z* = 2	0.32 × 0.28 × 0.19 mm

### Data collection

**Table:**

Bruker APEXII CCD diffractometer	11176 independent reflections
Radiation source: fine-focus sealed tube	6434 reflections with *I* > 2σ(*I*)
Graphite monochromator	*R*_int_ = 0.068
ω scans	θ_max_ = 30.5°, θ_min_ = 1.9°
Absorption correction: multi-scan (SADABS; Bruker, 2008)	*h* = −21→19
*T*_min_ = 0.953, *T*_max_ = 0.990	*k* = −10→18
24668 measured reflections	*l* = −27→27

### Refinement

**Table:**

Refinement on *F*^2^	Primary atom site location: structure-invariant direct methods
Least-squares matrix: full	Secondary atom site location: difference Fourier map
*R*[*F*^2^ > 2σ(*F*^2^)] = 0.065	Hydrogen site location: inferred from neighbouring sites
*wR*(*F*^2^) = 0.167	H-atom parameters constrained
*S* = 1.05	*w* = 1/[σ^2^(*F*_o_^2^) + (0.0601*P*)^2^] where *P* = (*F*_o_^2^ + 2*F*_c_^2^)/3
11176 reflections	(Δ/σ)_max_ = 0.001
455 parameters	Δρ_max_ = 0.73 e Å^−^^3^
4 restraints	Δρ_min_ = −0.73 e Å^−^^3^

### Special details

**Table:**

Experimental. moisture sensitive
Geometry. All esds (except the esd in the dihedral angle between two l.s. planes) are estimated using the full covariance matrix. The cell esds are taken into account individually in the estimation of esds in distances, angles and torsion angles; correlations between esds in cell parameters are only used when they are defined by crystal symmetry. An approximate (isotropic) treatment of cell esds is used for estimating esds involving l.s. planes.
Refinement. Refinement of F^2^ against ALL reflections. The weighted R-factor wR and goodness of fit S are based on F^2^, conventional R-factors R are based on F, with F set to zero for negative F^2^. The threshold expression of F^2^ > 2sigma(F^2^) is used only for calculating R-factors(gt) etc. and is not relevant to the choice of reflections for refinement. R-factors based on F^2^ are statistically about twice as large as those based on F, and R- factors based on ALL data will be even larger.

### Fractional atomic coordinates and isotropic or equivalent isotropic displacement parameters (Å^2^)

**Table:**

	*x*	*y*	*z*	*U*_iso_*/*U*_eq_	Occ. (<1)
P1	0.55033 (3)	0.44259 (4)	0.90237 (3)	0.01448 (12)	
Li1	0.3838 (2)	0.5809 (3)	0.9278 (2)	0.0213 (8)	
O1	0.47626 (9)	0.51925 (12)	0.89620 (8)	0.0205 (3)	
O2	0.59889 (9)	0.39677 (12)	0.97272 (7)	0.0194 (3)	
O3	0.50419 (9)	0.35531 (11)	0.84357 (7)	0.0151 (3)	
O4	0.62494 (9)	0.48621 (11)	0.86559 (7)	0.0153 (3)	
C1	0.55407 (13)	0.27551 (16)	0.82381 (11)	0.0152 (4)	
C2	0.58278 (13)	0.28822 (17)	0.76235 (11)	0.0164 (4)	
C3	0.63163 (14)	0.20661 (18)	0.74391 (12)	0.0208 (5)	
H3	0.653041	0.213333	0.703161	0.025*	
C4	0.64958 (15)	0.11659 (19)	0.78325 (12)	0.0236 (5)	
H4	0.684560	0.063138	0.770430	0.028*	
C5	0.61646 (15)	0.10444 (18)	0.84148 (12)	0.0228 (5)	
H5	0.627340	0.041411	0.867470	0.027*	
C6	0.56727 (14)	0.18322 (17)	0.86273 (11)	0.0177 (4)	
C7	0.55802 (14)	0.38231 (18)	0.71434 (11)	0.0191 (5)	
H7	0.521466	0.430478	0.735337	0.023*	
C8	0.49753 (15)	0.34927 (18)	0.63976 (11)	0.0224 (5)	
H8A	0.441843	0.315344	0.644160	0.034*	
H8B	0.531840	0.301087	0.618549	0.034*	
H8C	0.480482	0.410406	0.609224	0.034*	
C9	0.64346 (15)	0.43998 (19)	0.70861 (12)	0.0245 (5)	
H9A	0.680625	0.461248	0.756296	0.037*	
H9B	0.624670	0.501254	0.678475	0.037*	
H9C	0.679786	0.394355	0.687399	0.037*	
C10	0.52375 (15)	0.16555 (18)	0.92242 (12)	0.0227 (5)	
H10	0.507992	0.234472	0.938782	0.027*	
C11	0.43471 (17)	0.1045 (2)	0.89337 (14)	0.0424 (7)	
H11A	0.392397	0.143469	0.854634	0.064*	
H11B	0.406301	0.093086	0.931661	0.064*	
H11C	0.448332	0.037645	0.875163	0.064*	
C12	0.58768 (17)	0.1106 (2)	0.98601 (12)	0.0346 (6)	
H12A	0.644544	0.150448	1.003758	0.052*	
H12B	0.602071	0.041610	0.971541	0.052*	
H12C	0.557751	0.104102	1.023847	0.052*	
C13	0.71014 (13)	0.53203 (17)	0.89999 (11)	0.0162 (4)	
C14	0.78864 (13)	0.46933 (18)	0.91914 (11)	0.0184 (4)	
C15	0.87306 (14)	0.51964 (19)	0.94558 (12)	0.0228 (5)	
H15	0.927832	0.479722	0.958659	0.027*	
C16	0.87908 (15)	0.62598 (19)	0.95322 (12)	0.0244 (5)	
H16	0.937313	0.658343	0.971142	0.029*	
C17	0.79982 (14)	0.68476 (18)	0.93462 (11)	0.0221 (5)	
H17	0.804246	0.757661	0.940415	0.027*	
C18	0.71337 (14)	0.63940 (17)	0.90749 (11)	0.0173 (4)	
C19	0.78642 (14)	0.35270 (18)	0.91193 (12)	0.0209 (5)	
H19	0.721679	0.332070	0.886973	0.025*	
C20	0.84666 (16)	0.3149 (2)	0.86683 (13)	0.0292 (6)	
H20A	0.827961	0.349214	0.820239	0.044*	
H20B	0.839597	0.239936	0.860157	0.044*	
H20C	0.911081	0.331194	0.891170	0.044*	
C21	0.81396 (16)	0.3008 (2)	0.98530 (13)	0.0282 (5)	
H21A	0.770589	0.320787	1.011415	0.042*	
H21B	0.876017	0.322934	1.012125	0.042*	
H21C	0.812898	0.225598	0.979355	0.042*	
C22	0.62759 (15)	0.70588 (18)	0.88586 (11)	0.0223 (5)	
H22B	0.575884	0.662177	0.864565	0.027*	0.621 (4)
H22A	0.576724	0.666758	0.891825	0.027*	0.379 (4)
C23A	0.6322 (3)	0.7908 (4)	0.8323 (3)	0.0502 (8)	0.621 (4)
H23A	0.680451	0.840435	0.855262	0.075*	0.621 (4)
H23B	0.572964	0.826494	0.816431	0.075*	0.621 (4)
H23C	0.646298	0.759649	0.791122	0.075*	0.621 (4)
C24A	0.6142 (3)	0.7537 (4)	0.9543 (2)	0.0502 (8)	0.621 (4)
H24A	0.598060	0.699241	0.983290	0.075*	0.621 (4)
H24B	0.564707	0.805030	0.941420	0.075*	0.621 (4)
H24C	0.671314	0.787308	0.981735	0.075*	0.621 (4)
C23B	0.6103 (5)	0.7301 (8)	0.8058 (2)	0.0502 (8)	0.379 (4)
H23D	0.597206	0.665816	0.778271	0.075*	0.379 (4)
H23E	0.665026	0.762883	0.798606	0.075*	0.379 (4)
H23F	0.557789	0.777087	0.789744	0.075*	0.379 (4)
C24B	0.6330 (5)	0.8089 (5)	0.9258 (5)	0.0502 (8)	0.379 (4)
H24D	0.653973	0.796311	0.977356	0.075*	0.379 (4)
H24E	0.572053	0.841151	0.913091	0.075*	0.379 (4)
H24F	0.676336	0.855109	0.912496	0.075*	0.379 (4)
N1	0.31930 (12)	0.70375 (15)	0.85729 (9)	0.0211 (4)	
N2	0.24866 (12)	0.52388 (15)	0.88267 (10)	0.0219 (4)	
C25	0.35823 (15)	0.7887 (2)	0.83969 (12)	0.0256 (5)	
H25	0.421899	0.799352	0.862153	0.031*	
C26	0.31201 (17)	0.8618 (2)	0.79114 (13)	0.0289 (6)	
H26	0.343001	0.921075	0.781101	0.035*	
C27	0.21972 (16)	0.84689 (19)	0.75752 (12)	0.0265 (5)	
H27	0.185535	0.895560	0.723836	0.032*	
C28	0.17841 (15)	0.75913 (18)	0.77422 (12)	0.0224 (5)	
H28	0.114974	0.746887	0.751798	0.027*	
C29	0.22933 (14)	0.68876 (17)	0.82367 (11)	0.0176 (4)	
C30	0.18882 (14)	0.59147 (18)	0.84163 (11)	0.0185 (4)	
C31	0.09483 (15)	0.57103 (19)	0.81733 (12)	0.0244 (5)	
H31	0.053842	0.619906	0.788514	0.029*	
C32	0.06218 (17)	0.4787 (2)	0.83577 (13)	0.0306 (6)	
H32	−0.001704	0.463751	0.820308	0.037*	
C33	0.12327 (18)	0.4085 (2)	0.87683 (13)	0.0321 (6)	
H33	0.102711	0.343797	0.889342	0.039*	
C34	0.21567 (17)	0.4349 (2)	0.89937 (12)	0.0281 (5)	
H34	0.257714	0.387029	0.928266	0.034*	
C35	0.3720 (2)	0.5927 (2)	0.63362 (14)	0.0377 (7)	
C36	0.30727 (17)	0.5334 (2)	0.58269 (15)	0.0360 (6)	
H36	0.255945	0.504449	0.594142	0.043*	
C37	0.31846 (17)	0.5173 (2)	0.51625 (14)	0.0326 (6)	
H37	0.274745	0.477307	0.481915	0.039*	
C38	0.39274 (17)	0.5591 (2)	0.49934 (14)	0.0341 (6)	
H38	0.399718	0.547967	0.453268	0.041*	
C39	0.45656 (18)	0.6164 (2)	0.54835 (15)	0.0366 (6)	
H39	0.508186	0.644195	0.536704	0.044*	
C40	0.44553 (18)	0.6334 (2)	0.61384 (15)	0.0361 (6)	
H40	0.489572	0.674366	0.647204	0.043*	
C41	0.3599 (2)	0.6122 (3)	0.70541 (17)	0.0556 (9)	
H41A	0.360353	0.686795	0.714079	0.083*	
H41B	0.410290	0.579522	0.742045	0.083*	
H41C	0.301230	0.582977	0.707261	0.083*	

### Atomic displacement parameters (Å^2^)

**Table:**

	*U*^11^	*U*^22^	*U*^33^	*U*^12^	*U*^13^	*U*^23^
P1	0.0136 (2)	0.0155 (3)	0.0140 (3)	−0.0004 (2)	0.0035 (2)	−0.0007 (2)
Li1	0.0173 (17)	0.028 (2)	0.020 (2)	0.0027 (16)	0.0067 (15)	−0.0010 (16)
O1	0.0170 (7)	0.0212 (8)	0.0247 (9)	0.0034 (6)	0.0081 (6)	−0.0004 (7)
O2	0.0193 (7)	0.0245 (9)	0.0129 (7)	−0.0005 (6)	0.0020 (6)	0.0018 (6)
O3	0.0130 (7)	0.0152 (8)	0.0156 (7)	−0.0001 (6)	0.0020 (6)	−0.0017 (6)
O4	0.0123 (7)	0.0170 (8)	0.0154 (7)	−0.0029 (6)	0.0025 (6)	−0.0004 (6)
C1	0.0111 (9)	0.0150 (10)	0.0181 (11)	−0.0008 (8)	0.0018 (8)	−0.0030 (8)
C2	0.0141 (9)	0.0174 (11)	0.0160 (10)	−0.0028 (8)	0.0019 (8)	−0.0020 (8)
C3	0.0184 (10)	0.0250 (12)	0.0188 (11)	0.0013 (9)	0.0050 (9)	−0.0010 (9)
C4	0.0206 (11)	0.0227 (13)	0.0259 (12)	0.0043 (9)	0.0043 (9)	−0.0040 (10)
C5	0.0245 (11)	0.0157 (11)	0.0239 (12)	0.0008 (9)	0.0003 (9)	0.0022 (9)
C6	0.0170 (10)	0.0183 (11)	0.0151 (10)	−0.0030 (8)	0.0005 (8)	0.0012 (8)
C7	0.0190 (10)	0.0215 (12)	0.0166 (11)	0.0001 (9)	0.0050 (9)	0.0013 (9)
C8	0.0241 (11)	0.0240 (13)	0.0189 (11)	0.0006 (10)	0.0057 (9)	0.0033 (9)
C9	0.0265 (11)	0.0269 (13)	0.0207 (12)	−0.0052 (10)	0.0078 (9)	0.0024 (10)
C10	0.0294 (12)	0.0171 (11)	0.0230 (12)	−0.0031 (10)	0.0099 (10)	0.0040 (9)
C11	0.0343 (14)	0.061 (2)	0.0309 (15)	−0.0164 (14)	0.0086 (12)	0.0118 (14)
C12	0.0383 (14)	0.0443 (17)	0.0191 (13)	−0.0018 (13)	0.0052 (11)	0.0041 (11)
C13	0.0145 (10)	0.0200 (11)	0.0140 (10)	−0.0045 (8)	0.0041 (8)	−0.0017 (8)
C14	0.0158 (10)	0.0218 (12)	0.0171 (11)	0.0000 (9)	0.0039 (8)	0.0009 (9)
C15	0.0144 (10)	0.0269 (13)	0.0240 (12)	−0.0001 (9)	0.0009 (9)	−0.0013 (10)
C16	0.0178 (10)	0.0291 (14)	0.0243 (12)	−0.0088 (10)	0.0028 (9)	−0.0014 (10)
C17	0.0251 (11)	0.0189 (12)	0.0210 (12)	−0.0058 (9)	0.0047 (9)	−0.0008 (9)
C18	0.0175 (10)	0.0173 (11)	0.0172 (11)	−0.0011 (8)	0.0053 (8)	−0.0007 (8)
C19	0.0143 (10)	0.0206 (12)	0.0251 (12)	0.0007 (9)	0.0013 (9)	−0.0040 (9)
C20	0.0269 (12)	0.0307 (14)	0.0295 (14)	0.0029 (11)	0.0075 (10)	−0.0070 (11)
C21	0.0286 (12)	0.0240 (13)	0.0302 (14)	0.0026 (10)	0.0059 (10)	0.0032 (10)
C22	0.0193 (11)	0.0180 (12)	0.0275 (13)	−0.0004 (9)	0.0035 (9)	0.0003 (9)
C23A	0.0322 (15)	0.048 (2)	0.066 (2)	0.0117 (15)	0.0076 (14)	0.0011 (16)
C24A	0.0322 (15)	0.048 (2)	0.066 (2)	0.0117 (15)	0.0076 (14)	0.0011 (16)
C23B	0.0322 (15)	0.048 (2)	0.066 (2)	0.0117 (15)	0.0076 (14)	0.0011 (16)
C24B	0.0322 (15)	0.048 (2)	0.066 (2)	0.0117 (15)	0.0076 (14)	0.0011 (16)
N1	0.0174 (9)	0.0262 (11)	0.0183 (10)	−0.0017 (8)	0.0029 (7)	−0.0031 (8)
N2	0.0252 (10)	0.0229 (10)	0.0172 (10)	0.0027 (8)	0.0056 (8)	−0.0003 (8)
C25	0.0199 (11)	0.0327 (14)	0.0229 (12)	−0.0091 (10)	0.0041 (9)	−0.0054 (10)
C26	0.0353 (13)	0.0264 (14)	0.0259 (13)	−0.0088 (11)	0.0102 (11)	−0.0004 (10)
C27	0.0319 (13)	0.0229 (13)	0.0234 (12)	0.0012 (10)	0.0060 (10)	0.0020 (10)
C28	0.0197 (11)	0.0244 (13)	0.0218 (12)	0.0017 (9)	0.0039 (9)	−0.0001 (9)
C29	0.0161 (10)	0.0207 (11)	0.0160 (10)	0.0009 (9)	0.0047 (8)	−0.0018 (8)
C30	0.0192 (10)	0.0216 (12)	0.0138 (10)	0.0015 (9)	0.0033 (8)	−0.0026 (8)
C31	0.0203 (11)	0.0272 (13)	0.0241 (12)	−0.0005 (10)	0.0038 (9)	−0.0001 (10)
C32	0.0254 (12)	0.0349 (15)	0.0310 (14)	−0.0101 (11)	0.0074 (10)	−0.0024 (11)
C33	0.0429 (15)	0.0262 (14)	0.0283 (14)	−0.0120 (12)	0.0118 (12)	−0.0003 (11)
C34	0.0384 (14)	0.0222 (13)	0.0227 (12)	0.0009 (11)	0.0073 (10)	0.0046 (10)
C35	0.0534 (17)	0.0288 (15)	0.0322 (15)	0.0207 (13)	0.0146 (13)	0.0017 (12)
C36	0.0292 (13)	0.0405 (17)	0.0412 (16)	0.0106 (12)	0.0146 (12)	0.0075 (13)
C37	0.0274 (13)	0.0322 (15)	0.0331 (15)	0.0061 (11)	0.0009 (11)	0.0022 (12)
C38	0.0342 (14)	0.0330 (15)	0.0340 (15)	0.0113 (12)	0.0082 (11)	0.0113 (12)
C39	0.0356 (14)	0.0263 (15)	0.0473 (18)	0.0072 (12)	0.0111 (13)	0.0100 (12)
C40	0.0352 (14)	0.0201 (13)	0.0485 (18)	0.0050 (11)	0.0048 (13)	0.0028 (12)
C41	0.078 (2)	0.049 (2)	0.049 (2)	0.0113 (18)	0.0318 (18)	−0.0008 (16)

### Geometric parameters (Å, º)

**Table:**

Li1—O1	1.873 (4)	C21—H21B	0.9800
Li1—O2^i^	1.911 (4)	C21—H21C	0.9800
Li1—N1	2.147 (4)	C22—C23A	1.534 (3)
Li1—N2	2.119 (4)	C22—C24B	1.536 (4)
P1—O1	1.4795 (15)	C22—C24A	1.544 (3)
P1—O2	1.4846 (15)	C22—C23B	1.545 (4)
P1—O3	1.6198 (15)	C22—H22B	0.9599
P1—O4	1.6132 (14)	C22—H22A	0.9600
O3—C1	1.401 (2)	C23A—H23A	0.9800
O4—C13	1.406 (2)	C23A—H23B	0.9800
C1—C6	1.399 (3)	C23A—H23C	0.9800
C1—C2	1.405 (3)	C24A—H24A	0.9800
C2—C3	1.398 (3)	C24A—H24B	0.9800
C2—C7	1.517 (3)	C24A—H24C	0.9800
C3—C4	1.379 (3)	C23B—H23D	0.9800
C3—H3	0.9500	C23B—H23E	0.9800
C4—C5	1.384 (3)	C23B—H23F	0.9800
C4—H4	0.9500	C24B—H24D	0.9800
C5—C6	1.398 (3)	C24B—H24E	0.9800
C5—H5	0.9500	C24B—H24F	0.9800
C6—C10	1.520 (3)	N1—C25	1.341 (3)
C7—C9	1.531 (3)	N1—C29	1.349 (3)
C7—C8	1.543 (3)	N2—C34	1.334 (3)
C7—H7	1.0000	N2—C30	1.347 (3)
C8—H8A	0.9800	C25—C26	1.380 (3)
C8—H8B	0.9800	C25—H25	0.9500
C8—H8C	0.9800	C26—C27	1.380 (3)
C9—H9A	0.9800	C26—H26	0.9500
C9—H9B	0.9800	C27—C28	1.383 (3)
C9—H9C	0.9800	C27—H27	0.9500
C10—C12	1.516 (3)	C28—C29	1.389 (3)
C10—C11	1.528 (3)	C28—H28	0.9500
C10—H10	1.0000	C29—C30	1.488 (3)
C11—H11A	0.9800	C30—C31	1.395 (3)
C11—H11B	0.9800	C31—C32	1.382 (3)
C11—H11C	0.9800	C31—H31	0.9500
C12—H12A	0.9800	C32—C33	1.379 (4)
C12—H12B	0.9800	C32—H32	0.9500
C12—H12C	0.9800	C33—C34	1.388 (3)
C13—C18	1.396 (3)	C33—H33	0.9500
C13—C14	1.402 (3)	C34—H34	0.9500
C14—C15	1.398 (3)	C35—C40	1.390 (4)
C14—C19	1.515 (3)	C35—C36	1.408 (4)
C15—C16	1.384 (3)	C35—C41	1.492 (4)
C15—H15	0.9500	C36—C37	1.377 (4)
C16—C17	1.382 (3)	C36—H36	0.9500
C16—H16	0.9500	C37—C38	1.377 (4)
C17—C18	1.396 (3)	C37—H37	0.9500
C17—H17	0.9500	C38—C39	1.368 (4)
C18—C22	1.517 (3)	C38—H38	0.9500
C19—C20	1.527 (3)	C39—C40	1.359 (4)
C19—C21	1.530 (3)	C39—H39	0.9500
C19—H19	1.0000	C40—H40	0.9500
C20—H20A	0.9800	C41—H41A	0.9800
C20—H20B	0.9800	C41—H41B	0.9800
C20—H20C	0.9800	C41—H41C	0.9800
C21—H21A	0.9800		
			
O1—P1—O2	120.32 (9)	C19—C21—H21B	109.5
O1—P1—O4	110.33 (9)	H21A—C21—H21B	109.5
O2—P1—O4	109.25 (8)	C19—C21—H21C	109.5
O1—P1—O3	104.31 (8)	H21A—C21—H21C	109.5
O2—P1—O3	112.17 (9)	H21B—C21—H21C	109.5
O4—P1—O3	98.16 (8)	C18—C22—C23A	112.9 (2)
O1—Li1—O2^i^	120.5 (2)	C18—C22—C24B	115.8 (3)
O1—Li1—N2	116.4 (2)	C18—C22—C24A	107.8 (2)
O2^i^—Li1—N2	107.90 (17)	C23A—C22—C24A	110.5 (3)
O1—Li1—N1	110.41 (18)	C18—C22—C23B	106.1 (3)
O2^i^—Li1—N1	116.5 (2)	C24B—C22—C23B	108.0 (5)
N2—Li1—N1	77.49 (14)	C18—C22—H22B	108.5
P1—O1—Li1	152.40 (16)	C23A—C22—H22B	108.7
P1—O2—Li1^i^	140.35 (15)	C24A—C22—H22B	108.3
C1—O3—P1	123.55 (12)	C18—C22—H22A	109.0
C13—O4—P1	127.15 (12)	C24B—C22—H22A	108.7
C6—C1—O3	118.77 (18)	C23B—C22—H22A	109.0
C6—C1—C2	122.40 (19)	C22—C23A—H23A	109.5
O3—C1—C2	118.64 (19)	C22—C23A—H23B	109.5
C3—C2—C1	117.0 (2)	H23A—C23A—H23B	109.5
C3—C2—C7	120.03 (19)	C22—C23A—H23C	109.5
C1—C2—C7	122.83 (19)	H23A—C23A—H23C	109.5
C4—C3—C2	121.8 (2)	H23B—C23A—H23C	109.5
C4—C3—H3	119.1	C22—C24A—H24A	109.5
C2—C3—H3	119.1	C22—C24A—H24B	109.5
C3—C4—C5	119.8 (2)	H24A—C24A—H24B	109.5
C3—C4—H4	120.1	C22—C24A—H24C	109.5
C5—C4—H4	120.1	H24A—C24A—H24C	109.5
C4—C5—C6	121.2 (2)	H24B—C24A—H24C	109.5
C4—C5—H5	119.4	C22—C23B—H23D	109.5
C6—C5—H5	119.4	C22—C23B—H23E	109.5
C5—C6—C1	117.65 (19)	H23D—C23B—H23E	109.5
C5—C6—C10	120.7 (2)	C22—C23B—H23F	109.5
C1—C6—C10	121.48 (19)	H23D—C23B—H23F	109.5
C2—C7—C9	111.94 (17)	H23E—C23B—H23F	109.5
C2—C7—C8	109.67 (18)	C22—C24B—H24D	109.5
C9—C7—C8	110.52 (17)	C22—C24B—H24E	109.5
C2—C7—H7	108.2	H24D—C24B—H24E	109.5
C9—C7—H7	108.2	C22—C24B—H24F	109.5
C8—C7—H7	108.2	H24D—C24B—H24F	109.5
C7—C8—H8A	109.5	H24E—C24B—H24F	109.5
C7—C8—H8B	109.5	C25—N1—C29	117.0 (2)
H8A—C8—H8B	109.5	C25—N1—Li1	128.29 (18)
C7—C8—H8C	109.5	C29—N1—Li1	114.59 (18)
H8A—C8—H8C	109.5	C34—N2—C30	118.0 (2)
H8B—C8—H8C	109.5	C34—N2—Li1	125.9 (2)
C7—C9—H9A	109.5	C30—N2—Li1	115.35 (19)
C7—C9—H9B	109.5	N1—C25—C26	124.4 (2)
H9A—C9—H9B	109.5	N1—C25—H25	117.8
C7—C9—H9C	109.5	C26—C25—H25	117.8
H9A—C9—H9C	109.5	C25—C26—C27	118.5 (2)
H9B—C9—H9C	109.5	C25—C26—H26	120.8
C12—C10—C6	112.57 (19)	C27—C26—H26	120.8
C12—C10—C11	110.5 (2)	C26—C27—C28	118.1 (2)
C6—C10—C11	109.20 (19)	C26—C27—H27	120.9
C12—C10—H10	108.1	C28—C27—H27	120.9
C6—C10—H10	108.1	C27—C28—C29	120.2 (2)
C11—C10—H10	108.1	C27—C28—H28	119.9
C10—C11—H11A	109.5	C29—C28—H28	119.9
C10—C11—H11B	109.5	N1—C29—C28	121.8 (2)
H11A—C11—H11B	109.5	N1—C29—C30	115.93 (19)
C10—C11—H11C	109.5	C28—C29—C30	122.27 (19)
H11A—C11—H11C	109.5	N2—C30—C31	121.9 (2)
H11B—C11—H11C	109.5	N2—C30—C29	115.84 (18)
C10—C12—H12A	109.5	C31—C30—C29	122.3 (2)
C10—C12—H12B	109.5	C32—C31—C30	119.1 (2)
H12A—C12—H12B	109.5	C32—C31—H31	120.5
C10—C12—H12C	109.5	C30—C31—H31	120.5
H12A—C12—H12C	109.5	C33—C32—C31	119.3 (2)
H12B—C12—H12C	109.5	C33—C32—H32	120.3
C18—C13—C14	123.13 (19)	C31—C32—H32	120.3
C18—C13—O4	117.97 (18)	C32—C33—C34	118.1 (2)
C14—C13—O4	118.63 (19)	C32—C33—H33	120.9
C15—C14—C13	116.7 (2)	C34—C33—H33	120.9
C15—C14—C19	119.44 (19)	N2—C34—C33	123.6 (2)
C13—C14—C19	123.87 (18)	N2—C34—H34	118.2
C16—C15—C14	121.8 (2)	C33—C34—H34	118.2
C16—C15—H15	119.1	C40—C35—C36	117.5 (2)
C14—C15—H15	119.1	C40—C35—C41	121.9 (3)
C17—C16—C15	119.6 (2)	C36—C35—C41	120.5 (3)
C17—C16—H16	120.2	C37—C36—C35	119.9 (3)
C15—C16—H16	120.2	C37—C36—H36	120.0
C16—C17—C18	121.5 (2)	C35—C36—H36	120.0
C16—C17—H17	119.3	C36—C37—C38	120.3 (3)
C18—C17—H17	119.3	C36—C37—H37	119.9
C13—C18—C17	117.3 (2)	C38—C37—H37	119.9
C13—C18—C22	122.26 (19)	C39—C38—C37	120.5 (3)
C17—C18—C22	120.4 (2)	C39—C38—H38	119.7
C14—C19—C20	111.86 (19)	C37—C38—H38	119.7
C14—C19—C21	110.88 (18)	C40—C39—C38	119.5 (3)
C20—C19—C21	110.90 (19)	C40—C39—H39	120.3
C14—C19—H19	107.7	C38—C39—H39	120.3
C20—C19—H19	107.7	C39—C40—C35	122.2 (3)
C21—C19—H19	107.7	C39—C40—H40	118.9
C19—C20—H20A	109.5	C35—C40—H40	118.9
C19—C20—H20B	109.5	C35—C41—H41A	109.5
H20A—C20—H20B	109.5	C35—C41—H41B	109.5
C19—C20—H20C	109.5	H41A—C41—H41B	109.5
H20A—C20—H20C	109.5	C35—C41—H41C	109.5
H20B—C20—H20C	109.5	H41A—C41—H41C	109.5
C19—C21—H21A	109.5	H41B—C41—H41C	109.5
			
O2—P1—O1—Li1	−23.9 (4)	C14—C13—C18—C22	179.49 (19)
O4—P1—O1—Li1	−152.5 (3)	O4—C13—C18—C22	5.5 (3)
O3—P1—O1—Li1	103.0 (3)	C16—C17—C18—C13	0.1 (3)
O2^i^—Li1—O1—P1	39.6 (5)	C16—C17—C18—C22	−178.57 (19)
N2—Li1—O1—P1	−94.1 (3)	C15—C14—C19—C20	−55.2 (3)
N1—Li1—O1—P1	−179.9 (2)	C13—C14—C19—C20	124.0 (2)
O1—P1—O2—Li1^i^	−15.3 (3)	C15—C14—C19—C21	69.1 (3)
O4—P1—O2—Li1^i^	113.7 (2)	C13—C14—C19—C21	−111.6 (2)
O3—P1—O2—Li1^i^	−138.5 (2)	C13—C18—C22—C23A	−124.4 (3)
O1—P1—O3—C1	170.24 (15)	C17—C18—C22—C23A	54.2 (4)
O2—P1—O3—C1	−57.97 (17)	C13—C18—C22—C24B	153.2 (5)
O4—P1—O3—C1	56.74 (16)	C17—C18—C22—C24B	−28.2 (5)
O1—P1—O4—C13	99.30 (17)	C13—C18—C22—C24A	113.3 (3)
O2—P1—O4—C13	−35.08 (19)	C17—C18—C22—C24A	−68.1 (3)
O3—P1—O4—C13	−152.07 (16)	C13—C18—C22—C23B	−87.0 (4)
P1—O3—C1—C6	88.2 (2)	C17—C18—C22—C23B	91.6 (4)
P1—O3—C1—C2	−96.6 (2)	C29—N1—C25—C26	1.5 (3)
C6—C1—C2—C3	−4.5 (3)	Li1—N1—C25—C26	176.9 (2)
O3—C1—C2—C3	−179.41 (17)	N1—C25—C26—C27	−0.7 (4)
C6—C1—C2—C7	171.78 (18)	C25—C26—C27—C28	−0.1 (3)
O3—C1—C2—C7	−3.2 (3)	C26—C27—C28—C29	0.1 (3)
C1—C2—C3—C4	1.2 (3)	C25—N1—C29—C28	−1.5 (3)
C7—C2—C3—C4	−175.14 (19)	Li1—N1—C29—C28	−177.53 (19)
C2—C3—C4—C5	1.9 (3)	C25—N1—C29—C30	177.25 (19)
C3—C4—C5—C6	−2.0 (3)	Li1—N1—C29—C30	1.2 (3)
C4—C5—C6—C1	−1.1 (3)	C27—C28—C29—N1	0.7 (3)
C4—C5—C6—C10	174.4 (2)	C27—C28—C29—C30	−177.9 (2)
O3—C1—C6—C5	179.35 (17)	C34—N2—C30—C31	0.6 (3)
C2—C1—C6—C5	4.4 (3)	Li1—N2—C30—C31	−170.00 (19)
O3—C1—C6—C10	3.9 (3)	C34—N2—C30—C29	−179.11 (19)
C2—C1—C6—C10	−171.00 (19)	Li1—N2—C30—C29	10.3 (3)
C3—C2—C7—C9	−63.0 (3)	N1—C29—C30—N2	−7.6 (3)
C1—C2—C7—C9	120.9 (2)	C28—C29—C30—N2	171.1 (2)
C3—C2—C7—C8	60.0 (2)	N1—C29—C30—C31	172.6 (2)
C1—C2—C7—C8	−116.1 (2)	C28—C29—C30—C31	−8.7 (3)
C5—C6—C10—C12	44.7 (3)	N2—C30—C31—C32	−0.1 (3)
C1—C6—C10—C12	−140.0 (2)	C29—C30—C31—C32	179.6 (2)
C5—C6—C10—C11	−78.4 (3)	C30—C31—C32—C33	−0.9 (4)
C1—C6—C10—C11	96.8 (3)	C31—C32—C33—C34	1.4 (4)
P1—O4—C13—C18	−92.1 (2)	C30—N2—C34—C33	−0.1 (3)
P1—O4—C13—C14	93.6 (2)	Li1—N2—C34—C33	169.4 (2)
C18—C13—C14—C15	−1.2 (3)	C32—C33—C34—N2	−0.9 (4)
O4—C13—C14—C15	172.78 (18)	C40—C35—C36—C37	0.2 (4)
C18—C13—C14—C19	179.5 (2)	C41—C35—C36—C37	179.0 (3)
O4—C13—C14—C19	−6.5 (3)	C35—C36—C37—C38	0.0 (4)
C13—C14—C15—C16	0.6 (3)	C36—C37—C38—C39	0.4 (4)
C19—C14—C15—C16	180.0 (2)	C37—C38—C39—C40	−1.0 (4)
C14—C15—C16—C17	0.3 (3)	C38—C39—C40—C35	1.2 (4)
C15—C16—C17—C18	−0.6 (3)	C36—C35—C40—C39	−0.8 (4)
C14—C13—C18—C17	0.8 (3)	C41—C35—C40—C39	−179.6 (3)
O4—C13—C18—C17	−173.17 (17)		

Symmetry code: (i) −*x*+1, −*y*+1, −*z*+2.

### Hydrogen-bond geometry (Å, º)

*Cg*1 is the centroid of N1/C25–C29, *Cg*2 is the centroid of N2/C30–C34, *Cg*3 is the centroid of C1–C6 and *Cg*5 is the centroid of C35–C40.

**Table:**

*D*—H···*A*	*D*—H	H···*A*	*D*···*A*	*D*—H···*A*
C28—H28···O3^ii^	0.95	2.60	3.295 (3)	130
C8—H8*C*···*Cg*5	0.98	2.60	3.502 (3)	152
C19—H19···*Cg*3	1.00	2.73	3.630 (2)	150
C41—H41*A*···*Cg*1	0.98	2.83	3.450 (4)	122
C26—H26···*Cg*2^ii^	0.95	2.94	3.605 (3)	128
C31—H31···*Cg*3^ii^	0.95	2.69	3.591 (3)	159

Symmetry code: (ii) −*x*+1/2, *y*+1/2, −*z*+3/2.
