# Supplementary material for: Bis[μ-bis­(2,6-diiso­propyl­phen­yl) phosphato-κ2 O:O′]bis­[(2,2′-bi­pyridine-κ2 N,N′)lithium] toluene disolvate and its catalytic activity in ring-opening polymerization of ∊-caprolactone and l-dilactide
Source: Acta Crystallogr E Crystallogr Commun. 2019 May 21;75(Pt 6):848–53. doi: 10.1107/S2056989019006960 (PMC6658944; doi:10.1107/S2056989019006960)
Supplement: Supplementary file 4 [file e-75-00848-sup4.doc]

**Bis[bis(2,6-diisopropylphenyl)phosphato-μ_2_-1κO:2κO']-bis(2,2'-bipyridine-κ^2^N,N')-dilithium toluene disolvate and its catalytic activity in ring-opening polymerisation of ε-caprolactone and L-dilactide.**

Alexey E. Kalugin,**^a,b^** Pavel D. Komarov,**^b^** Mikhail E. Minyaev,**^b^*** Konstantin A. Lyssenko,**^c,d^** Dmitrii M. Roitershtein**^b,e^** and Ilya E. Nifant'ev.**^b,c^**

**Supporting Information**

**^a^**Moscow Institute of Physics and Technology, Department of Biological and Medical Physics, 9 Institutskiy Per., Dolgoprudny, Moscow Region, 141701, Russian Federation.

**^b^**A.V. Topchiev Institute of Petrochemical Synthesis, Russian Academy of Sciences, 29 Leninsky prospect, 119991, Moscow, Russia.

**^c^**Chemistry Department, M.V. Lomonosov Moscow State University, 1 Leninskie Gory Str., Building 3, Moscow 119991, Russian Federation.

**^d^**G.V. Plekhanov Russian University of Economics, 36 Stremyanny Per., Moscow, 117997, Russian Federation.

**^e^**N.D. Zelinsky Institute of Organic Chemistry, Russian Academy of Sciences, 47 Leninsky Prospect, Moscow, 119991, Russian Federation.


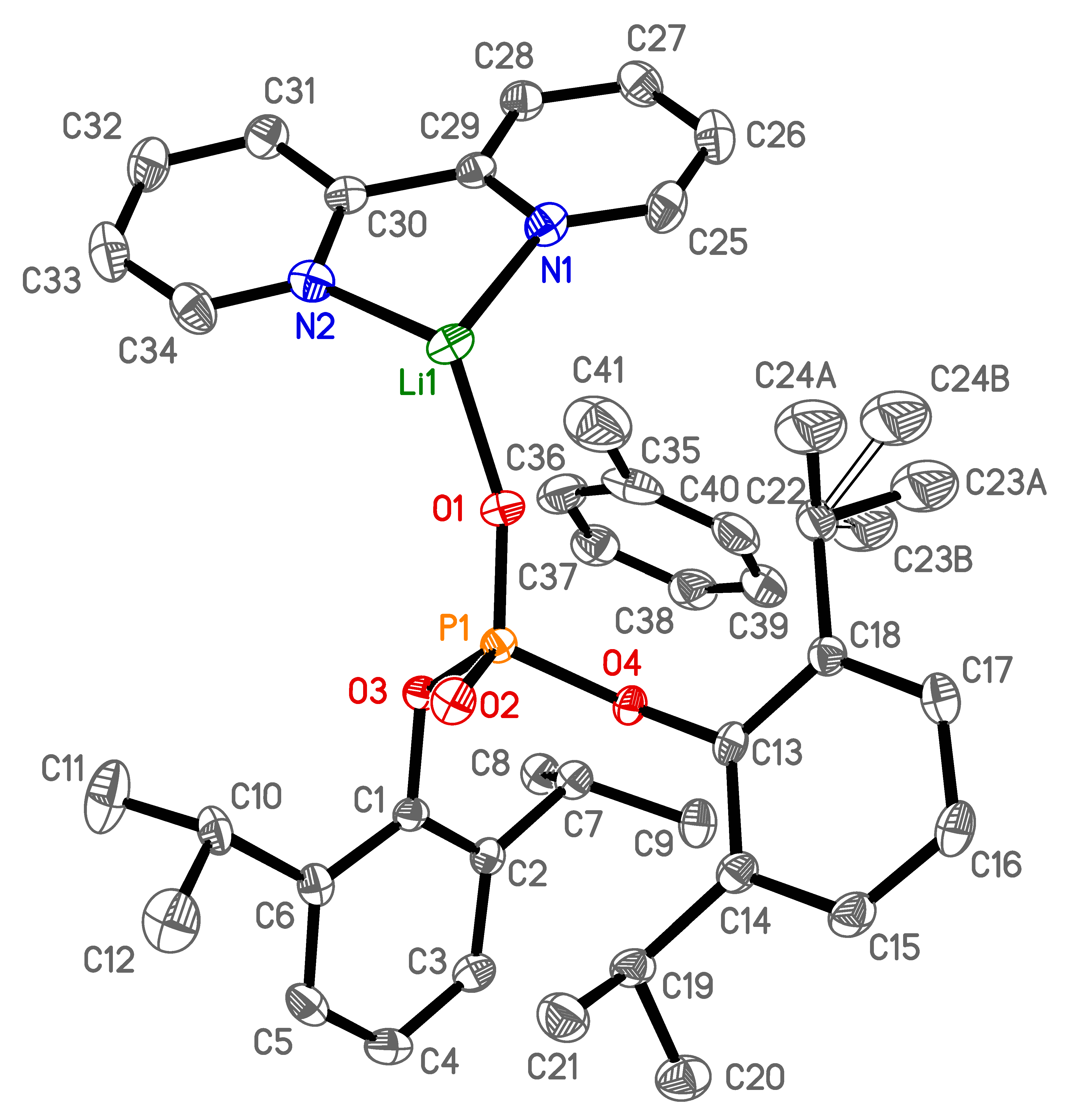


**Figure S1.** The asymmetric unit of {Li_2_(bipy)_2_[(2,6-^i^Pr_2_C_6_H_3_-O)_2_PO_2_]_2_}(toluene)_2_. Displacement ellipsoids are set to the 50% probability level. Symmetry code: (i) -x+1, -y+1, -z+2.


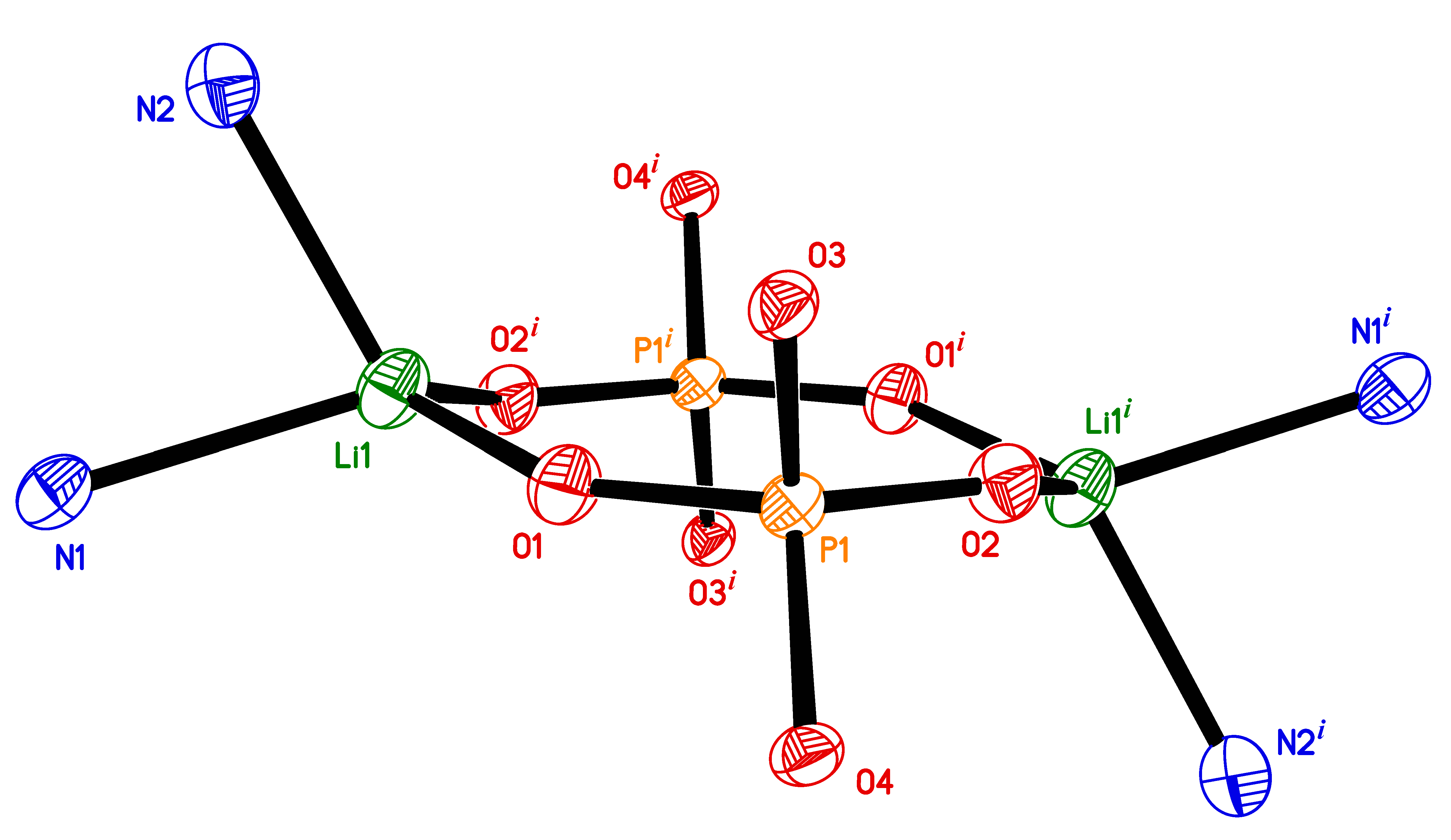


**Figure S2.** Core atoms in the complex {Li_2_(bipy)_2_[(2,6-^i^Pr_2_C_6_H_3_-O)_2_PO_2_]_2_}. Displacement ellipsoids are set to the 50% probability level. Symmetry code: (i) -x+1, -y+1, -z+2.


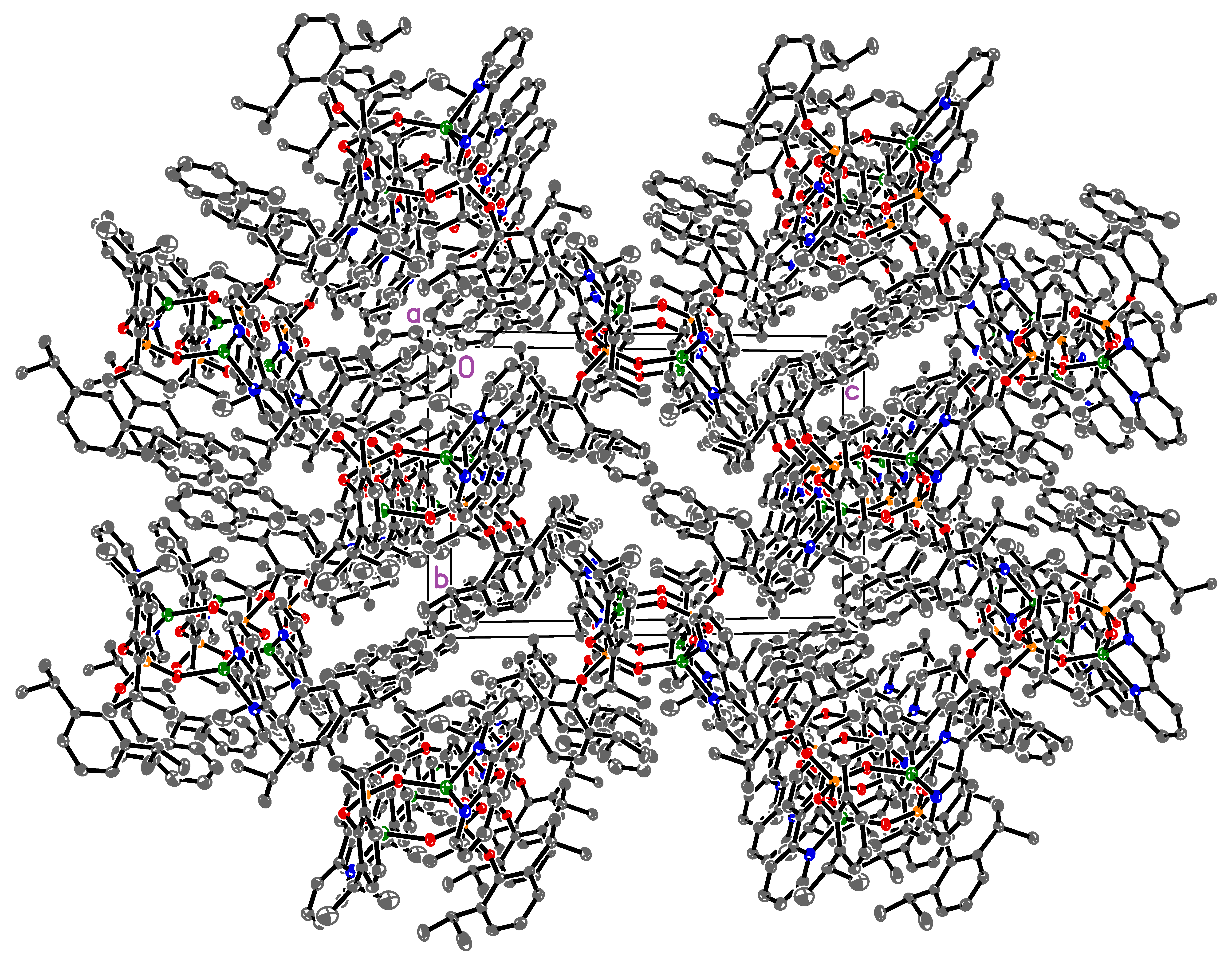


**Figure S3.** The packing plot of {Li_2_(bipy)_2_[(2,6-^i^Pr_2_C_6_H_3_-O)_2_PO_2_]_2_}(toluene)_2_ parallel to *bc* plane. Disorder is not shown.


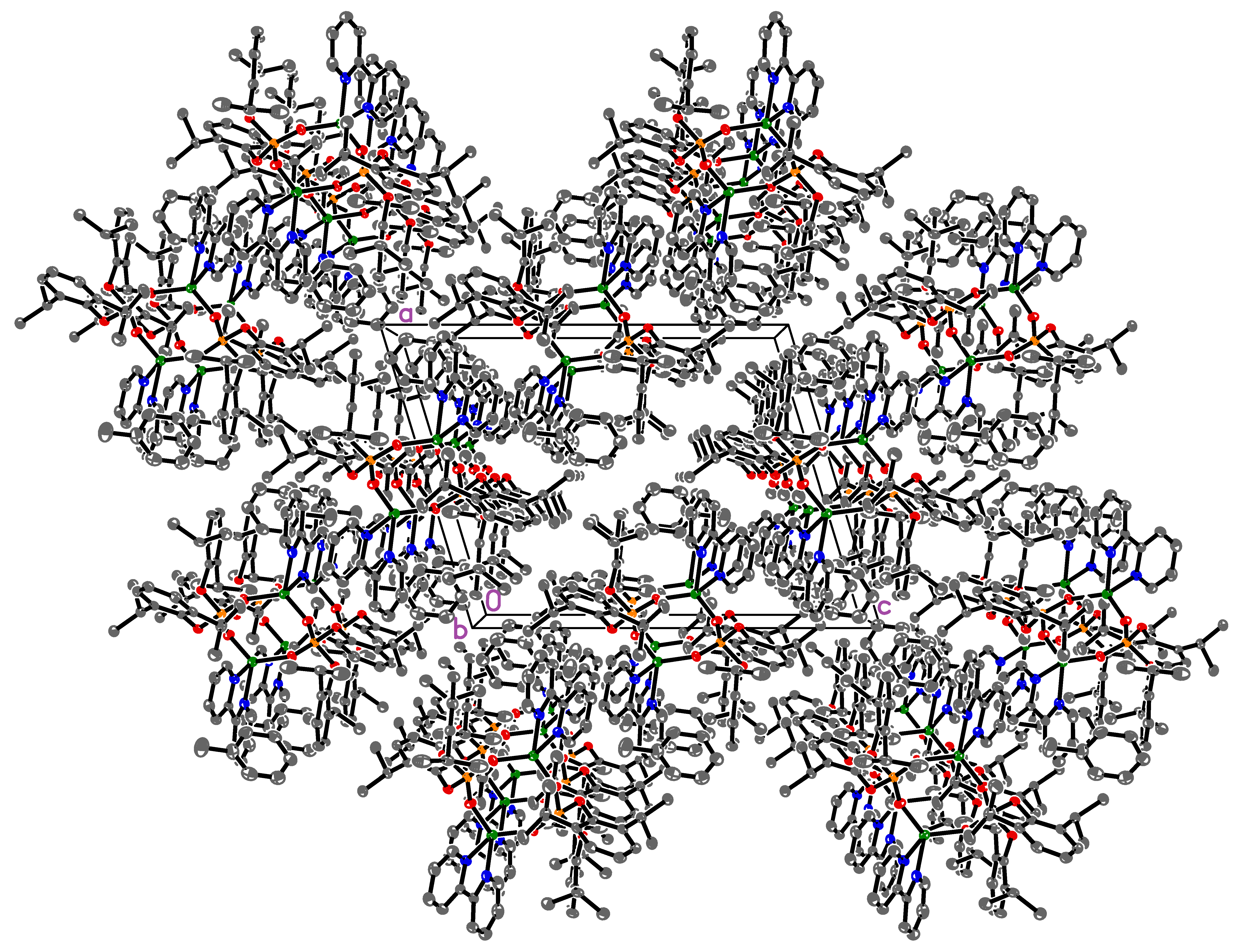


**Figure S4.** The packing plot of {Li_2_(bipy)_2_[(2,6-^i^Pr_2_C_6_H_3_-O)_2_PO_2_]_2_}(toluene)_2_ parallel to *ac* plane. Disorder is not shown.


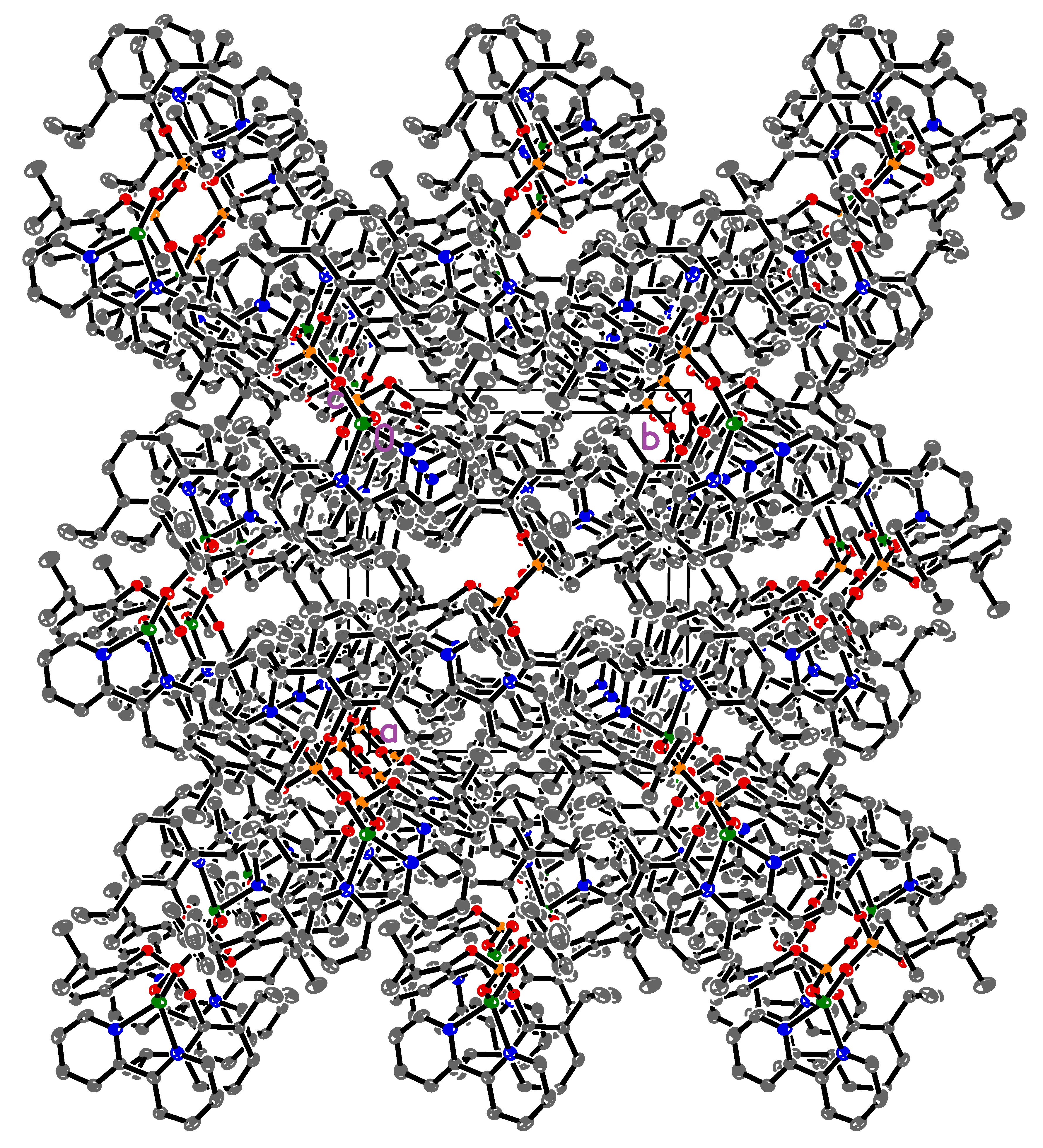


**Figure S5.** The packing plot of {Li_2_(bipy)_2_[(2,6-^i^Pr_2_C_6_H_3_-O)_2_PO_2_]_2_}(toluene)_2_ parallel to *ab* plane. Disorder is not shown.
